# Two-Photon Compatibility and Single-Voxel, Single-Trial Detection of Subthreshold Neuronal Activity by a Two-Component Optical Voltage Sensor

**DOI:** 10.1371/journal.pone.0041434

**Published:** 2012-08-03

**Authors:** Ann E. Fink, Kevin J. Bender, Laurence O. Trussell, Thomas S. Otis, David A. DiGregorio

**Affiliations:** 1 Unit of Dynamic Neuronal Imaging, Department of Neuroscience, Paris, France; 2 Genes, Synapses and Cognition, CNRS URA 2182, Institut Pasteur, Paris, France; 3 Vollum Institute and Oregon Hearing Research Center, Oregon Health and Science University, Portland, Oregon, United States of America; 4 Department of Neurobiology, David Geffen School of Medicine, University of California Los Angeles, Los Angeles, California, United States of America; University of New South Wales, Australia

## Abstract

Minimally invasive measurements of neuronal activity are essential for understanding how signal processing is performed by neuronal networks. While optical strategies for making such measurements hold great promise, optical sensors generally lack the speed and sensitivity necessary to record neuronal activity on a single-trial, single-neuron basis. Here we present additional biophysical characterization and practical improvements of a two-component optical voltage sensor (2cVoS), comprised of the neuronal tracer dye, DiO, and dipicrylamine (DiO/DPA). Using laser spot illumination we demonstrate that membrane potential-dependent fluorescence changes can be obtained in a wide variety of cell types within brain slices. We show a correlation between membrane labeling and the sensitivity of the magnitude of fluorescence signal, such that neurons with the brightest membrane labeling yield the largest ΔF/F values per action potential (AP; ∼40%). By substituting a blue-shifted donor for DiO we confirm that DiO/DPA works, at least in part, via a Förster resonance energy transfer (FRET) mechanism. We also describe a straightforward iontophoretic method for labeling multiple neurons with DiO and show that DiO/DPA is compatible with two-photon (2P) imaging. Finally, exploiting the high sensitivity of DiO/DPA, we demonstrate AP-induced fluorescence transients (fAPs) recorded from single spines of hippocampal pyramidal neurons and single-trial measurements of subthreshold synaptic inputs to granule cell dendrites. Our findings suggest that the 2cVoS, DiO/DPA, enables optical measurements of trial-to-trial voltage fluctuations with very high spatial and temporal resolution, properties well suited for monitoring electrical signals from multiple neurons within intact neuronal networks.

## Introduction

A long-standing goal in neuroscience is to record all neural activity within particular circuits. This benchmark requires methodology to detect APs in parallel from individual neurons with sufficiently high signal-to-noise ratio (SNR) such that in single trials every AP can be recorded. At present this is not possible, but it is widely recognized that optical approaches provide a viable solution to this significant technical challenge [1]. Optical reporters of membrane voltage have recently been used to measure APs in compartments as small as single spines [2], [3], [4] and terminals [5]. Even at this early stage of development, optical approaches have revolutionized the study of neuronal circuit physiology [6], [7].

In recent years there has been significant progress in improving the performance of voltage sensitive dyes (VSDs). The most widely used VSDs are the electrochromic styryl dyes [2], [3], [8], [9], [10], [11], [12]. These dyes can generate large signals in distal subcompartments of large neurons, within acute brain slices. However, application of these dyes is restricted to large neurons and labeling methods are challenging, requiring patch clamp techniques and long loading times [6]. An alternative to electrochromic styryl dyes are FRET-based 2cVoS [13]. Voltage sensing with 2cVoS results from a FRET interaction between a donor and an acceptor component, one of which translocates within the changing electric field across the lipid bilayer. We recently introduced a 2cVoS pair consisting of DiO, a neuronal tracer dye as a FRET donor and DPA as a non-fluorescent acceptor [14]. This 2cVoS exhibits one of the largest relative changes per millivolt of membrane potential change (i.e., sensitivity) among rapid (submillisecond) voltage indicators. When coupled with laser illumination and diffraction-limited excitation volumes, AP- induced fluorescence transients (fAPs) could be detected in single trials. Large fAP signals (∼20% ΔF/F) with high SNRs (>6) were observed in Purkinje cell bodies within cerebellar slices, while signals from their axons were significantly larger (>30% ΔF/F). More recently, genetically encoded indicators [15] and photo-induced electron transfer-based organic sensors [16] show promising improvements in both speed and sensitivity.

In this study we further characterize DiO/DPA, describing conditions for optimizing the performance and limiting the shortcomings of this method. We show DiO/DPA detection of single APs and EPSPs in small dendritic subcompartments of different types of neurons. We report a single neuron iontophoretic method for rapidly and reliably targeting DiO labeling to multiple neurons. We also show that DiO/DPA is amenable to 2P excitation, making it possible to record from multiple neurons deep within brain tissue. Finally, we take advantage of the combination of speed and sensitivity of the DiO-DPA 2cVoS to perform single-trial detection of back-propagating APs (bAPs) in dendritic spines of CA1 pyramidal neurons and subthreshold EPSPs in somatic and dendritic compartments of cerebellar granule cells. Strikingly, small neurons showed fAP peak amplitudes of >40% ΔF/F, potentially due to strong membrane-specific labeling. These findings show that DiO/DPA is one of the most sensitive and versatile submillisecond optical techniques. It can be implemented in a straightforward manner from a wide array of neuronal types, while generating large SNRs sufficient for probing excitability in either neuronal subcompartments or intact neuronal circuits.

## Materials and Methods

### Slice Preparation

Experimental procedures were approved by the Oregon Health & Science University Institutional Animal Care and Use Committee (IACUC), the University of California IACUC, and the veterinary staff of the Institut Pasteur. Parasagittal cerebellar slices (200 µm) or coronal hippocampal slices (300 µm) were prepared using a Leica VT1200S tissue slicer, according to standard methods, from 30–60 day old adult male and female CB6 mice. Slicing was performed in ice-cold artificial cerebrospinal fluid (ACSF), and slices were maintained at 34°C for 30 minutes in the slicing solution before being transferred to recording solution where they were brought to room temperature and kept for several hours. Slicing solution contained (in mM):85 NaCl, 2.5 KCl, 1.25 NaH_2_PO_4_, 25 NaHCO_3_, 0.5 CaCl_2_, 7 MgCl_2_, 25 glucose, 75 sucrose and 0.5 L-ascorbic acid. Recording solution for cerebellar slices contained (in mM): 125 NaCl, 2.5 KCl, 1.25 NaH_2_PO_4_, 25 NaHCO_3_, 2 CaCl_2_, 1.3 MgCl_2_, 25 glucose and 0.5 L-ascorbic acid. Recording solution for hippocampal slices contained (in mM): 125 NaCl, 2.5 KCl, 1.25 NaH_2_PO_4_, 25 NaHCO_3_, 2 CaCl_2_, 1.3 MgCl_2_, 15 glucose and 0.5 L-ascorbic acid.

For iontophoretic loading experiments in cortical pyramidal cells, “across row” slices [17] through primary somatosensory cortex were made in P17-23 CBA mice. Recordings were made from cortical layer 2/3 and layer 5b pyramidal neurons. All experiments were done at 32–34°C, except for the multiple-cell loading which was performed at 21°C. The external solutions for somatosensory cortex are as follows (both bubbled with 5% CO_2_/95%O_2_). Slicing solution: 87 mM NaCl, 25 mM, NaHCO_3_, 25 mM glucose, 75 mM sucrose, 2.5 mM KCl, 1.25 mM NaH_2_PO_4_, 0.5 mM CaCl_2,_ and 7 mM MgCl_2_ at 4°C. Recording solution: 130 NaCl, 3 KCl, 2.4 CaCl_2_, 1.3 MgSO_4_, 1.2 KH2PO_4_, 20 NaHCO_3_, 3 Na-HEPES, and 10 glucose.

### Patch-clamp Recording

Patch pipettes were backfilled with a KMeSO_3_-based patch pipette solutions including (in mM): 110 (granule cells) or 115 (Purkinje/CA1 pyramidal cells) KMeSO_3_, 10.3 Na^+^ (NaOH, Na-ATP, Na-GTP), 4.6 MgCl_2_, 40 HEPES, 1 EGTA, 4 mM ATP, 0.3 GTP, osmolarity  = 280–285 (granule) or 290–295 (pyramidal/Purkinje), pH: 7.3. All cells were corrected offline for a -7 mV liquid junction potential. Currents were injected to hold granule cells at ∼82 mV, and Purkinje and pyramidal cells at ∼72 mV. 20 µM Alexa-594 (Invitrogen) was also added to the internal solutions to verify neuronal morphology and loading of the membrane tracer dye. Internal solutions for pyramidal cells in somatosensory cortex included (in mM): 113 K-Gluconate, 9 HEPES, 4.5 MgCl_2_, 0.1 EGTA, 14 Tris_2_-phosphocreatine, 4 Na_2_-ATP, 0.3 tris-GTP; ∼290 mOsm, pH: 7.2–7.25. For electrical recordings of membrane voltage, 2 µM DPA (City Chemicals), diluted from a 20 mM stock in DMSO, was bath-applied in normal external solution. Voltage-clamp and current-clamp recordings were acquired at 25 kHz and filtered at 10 kHz using a MultiClamp 700B patch amplifier (Molecular Devices).

### Confocal Imaging and Optical Spot Measurements

Confocal images and spot detection were performed on a single-photon confocal microscope (Prairie Technologies) as described previously [14]. Confocal illumination was performed with 488 nm (PhoxX, Omicron) and 561 nm (Cobalt) diode lasers. Laser intensity and temporal gating were implemented using an AOTF for 561 nm and by direct modulation for 488 nm. In the latter case, a 488/10 nm clean-up filter (Chroma) and absorptive neutral density filters were also used. 375 and 405 nm laser illumination was performed with Deepstar diode lasers (Omicron), whose intensity was directly modulated, then spectrally filtered using a 375/20 nm and 405/20 nm clean-up filter, respectively (Chroma). Laser light was spatially filtered by a single mode fiber whose output was collimated and adjusted to overfill a 60× objective (1.0 N.A., Nikon) or a 100× objective (1.1 N.A., Nikon). In the specimen plane, a diffraction limited illumination spot was formed (width ∼300 nm). A 540/80 nm band pass filter was used to maximize emission for DiO (DiO_16_, Invitrogen), and a 450/50 nm band pass filter was used for DiB (Biotium) emission. DiO fluorescence detection was performed with a gallium-arsenide-phosphide photo-cathode photomultiplier tube (PMT, H7442, Hamamatsu). Point measurements of membrane voltage were obtained without the detection pinhole unless otherwise stated. Fluorescence signals were detected from structures within 30 µm of the slice surface to avoid prohibitive levels of light scattering by surrounding tissue. Granule cells were recorded up to a depth of 20 µm from the slice surface. Fluorescence point measurements were filtered at 10 kHz, then acquired at 25 kHz along with electrophysiological measurements using an analogue to digital conversion card (NI-DAQ 6053, National Instruments) that was controlled by IGORPro software (Wavemetrics) running the Neuromatic acquisition package (www.neuromatic.thinkrandom.com). DiO/DPA produces a decrease in fluorescence upon depolarization; therefore, except where indicated, all relative fluorescence transients were inverted (multiplication by −1) in order to match the depolarization convention of electrophysiology.

### 2P Imaging and Spot-detection

2P imaging was performed using titanium-sapphire lasers (Ultra I, Coherent or Mai-Tai HP DeepSee, Spectra Physics). In experiments where wavelength was varied, laser energy delivered to the scan head was monitored after the Pockels cell (Conoptics) and maintained at a constant level. For optical voltage measurements, DiO/DPA was imaged with epi- and transfluorescence gallium-arsenide-phosphide photo-cathode PMTs (H8224 Hamamatsu, cooled to 5°C) using 920 or 940 nm excitation. Cells were typically located 20–100 µm from the slice surface. DiO/DPA fluorescence changes were acquired in point scan mode at 5 kHz, and then filtered offline with a Gaussian filter corresponding to 0.5 kHz (IGORPro).

### Application of DPA

Stock solutions of DPA were prepared at 20 mM in DMSO and stored at room temperature. Before optical recordings, slices were pre-incubated for at least 45 min in external solution containing 2 µM DPA (0.01% DMSO). Unless indicated otherwise, all dye labeling and optical measurements were also conducted using 2 µM DPA, a concentration found to produce robust signals without significant effects on AP firing [14]. The presence of DPA in the bathing solution at all times also ensures the recording from healthy cells, because depolarized membrane potentials resulted in quenched DiO fluorescence by DPA.

### Patch-labeling with DiO

Stock solutions of DiO were prepared at 1.5 mM in DMSO. We then diluted the stock fresh every day in internal solution to 0.05–0.2% (0.75–3 µM final) for cell-attached labeling and to 0.1% for whole-cell recordings. DiO-containing internal solution was stored at room temperature and vortexed just prior to backfilling each patch pipette. In some cases the DiO-containing internal solution was prepared multiple times in a day by adding DiO stock to a pipette solution maintained on ice in order to minimize hydrolysis of ATP. For brief on-cell loading, cells were held in cell-attached mode with a DiO-containing internal solution for up to ∼5 min, or until whole-cell configuration occurred spontaneously. The pipette was then withdrawn in the same way as when pulling an outside-out patch.

To obtain adequate DiO loading, we noted that it was important to have visible dye accumulations at the very tip of the pipette [14]. The brief spontaneous whole-cell access usually observed during dye loading also appeared to correlate with good labeling, presumably because enhanced contact of the membrane with the accumulations of DiO at the pipette tip. Also crucial for seal formation and dye labeling was the size and shape of the pipette tip. Steeply tapered patch-pipettes were pulled from thick-walled borosilicate glass on a Sutter Instruments P-1000 puller. Before performing dye loading experiments, pipette tips were polished using a Narishige MF-900 Microforge until a visible change in shape was observed. Final resistances of reliable loading pipettes were generally high (7–11 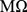 for pyramidal or Purkinje cells, 9–13 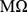 for granule cells).

### Iontophoretic Labeling

Neuronal membranes were labeled with DiO via iontophoretic current by positioning a 15 MΩ pipette (measured with internal solution in pipette) close to the soma surface and passing outward current (1–10 nA, 0.2–1s) to expel the positively charged DiO. For these experiments, instead of DMSO, DiO was dissolved in ethanol at saturating concentrations. These solutions were sonicated, centrifuged with a minifuge, and the supernatant was collected for use. Heating this solution to 50°C also helped maximize dye solubility. Best results were obtained with fresh pipettes. To achieve a high success rate of cell loading that allowed for subsequent imaging of physiological activity (>50%), it was imperative to monitor DiO ejection, stopping current pulses at the first indication of membrane loading. This procedure was performed using simultaneous 2P imaging and laser scanning Dodt contrast. Cells were often patched before DiO loading, but whole-cell recordings could also be obtained after loading procedures. All loading and recording was performed in the presence of 2 µM DPA.

### Minimal Stimulation of Optical Synaptic Responses in Cerebellar Granule cells

Minimal stimulation of mossy fiber-granule cell synapses was performed in a similar manner to that described previously [18]. Extracellular stimulation was performed using a stimulation isolation unit (Digitimer). To search for mossy fiber inputs, a train of 50-Hz or 100-Hz synaptic stimulation was delivered through a patch pipette filled with external solution, which was placed in various locations within ∼15 µm of the granule cell soma, while recording the optical signal from the granule cell soma. As soon as a synaptic input was observed, stimulation strength was lowered gradually until the response disappeared. After this, the stimulation was increased again by up to 15 V to obtain a reliable unitary input. When decreasing stimulation strength, we never observed a corresponding gradual decrease in amplitude of the subthreshold optical response; nor did we obtain any additional increase in ΔF/F of the optical response by increasing stimulation strength, indicating that we were stimulating single mossy fibers.

### Data Analysis

**Figure 1:**
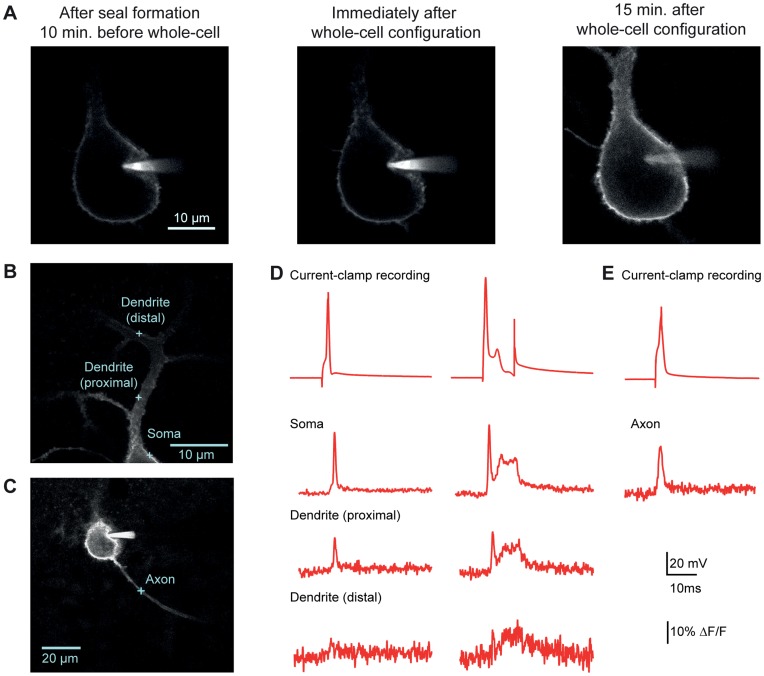
Optical voltage measurements from subcompartments of a cerebellar Purkinje cell. A. Confocal images of DiO labeling in a Purkinje cell acquired upon sealing with the DiO-filled pipette (left), upon rupturing the membrane (center) and 15 minutes after attaining whole-cell configuration (right). **B.** Confocal image of a Purkinje cell patch-loaded with DiO. Blue crosses indicate spots where optical measurements were taken in panels D and E. **C.** Confocal image of the same Purkinje cell with a cross indicating the spot where an axonal measurement was taken. **D.** Single APs (left) and complex-like spikes (right) recorded either electrophysiologically at the soma (top trace) or optically from different regions within the same cell. As indicated, some optical traces in this and subsequent figures are averaged with number of traces indicated in parentheses. Displayed here are AP and complex spike-like signals, respectively, in the soma (15, 9), in the proximal dendrite (12,12), and in the distal dendrite (8,9). **E.** The upper trace is the soma electrical recording of membrane voltage, and the trace below is an average of 6 fAPs recorded in the axon.

Analysis of electrical and optical (spot detection) signals was also carried out using IGOR Pro and Neuromatic software. To estimate the membrane-specificity of DiO labeling, fluorescence images were analyzed using the ImageJ software package (http://rsbweb.nih.gov/ij/). 1 µm-wide intensity profiles were calculated from single images where the somata were bisected such that the profile included strong membrane labeling (see Figure 1A-C).

**Figure 2:**
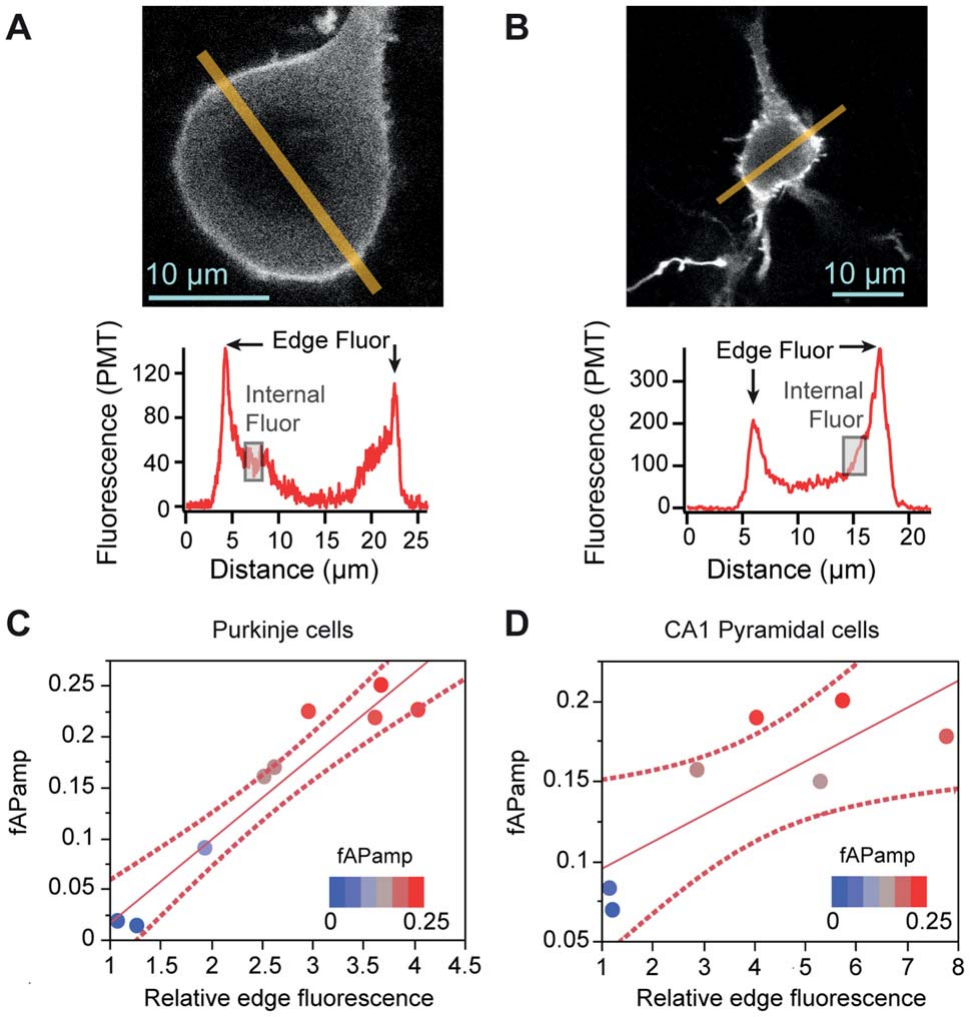
Membrane-specificity of DiO labeling predicts larger ΔF/F per AP. A-B. Sample confocal images with 1 µm-wide fluorescence line profiles taken from a Purkinje cell (**A**) and a hippocampal CA1 pyramidal cell (**B)**. Regions of edge fluorescence are indicated by arrows and sample regions of internal fluorescence are indicated by shaded boxes. **C-D.** Linear fits (solid line) of fAP amplitude vs. ”relative edge fluorescence” with 95% confidence intervals denoted by dashed lines. Circles represent individual data points, with blue points representing the lowest fAP amplitudes and the red points representing the highest fAP amplitudes. Significant correlations are observed in both Purkinje cells (**C**) and Pyramidal cells (**D**).

Prior to analysis, spot-detected optical signals were filtered offline at 2 kHz. The PMT dark current (region prior to laser illumination) was first subtracted from the traces. We then subtracted background fluorescence, which was estimated from the average fluorescence produced by a laser pulse illumination (same duration as for traces with stimuli) at a spot location roughly 10 µm away from the cell. In confocal measurements without the confocal pinhole, the background and cell fluorescence was first corrected for sublinearities in photon counting according to Bradley et al. [14]. ΔF/F was calculated from the ratio of the change in fluorescence relative to the resting fluorescence just before the stimulus. ΔF/F per 100 mV was calculated only for somatic fluorescence transients by dividing the peak ΔF/F by the peak membrane potential of electrically recorded APs minus the resting membrane potential, then multiplying by 100 mV. Dye photobleaching (approximately 10–20% of initial fluorescence values) was corrected by subtracting the double exponential fit of an average fluorescence trace without stimulus (see [14]).

**Figure 3:**
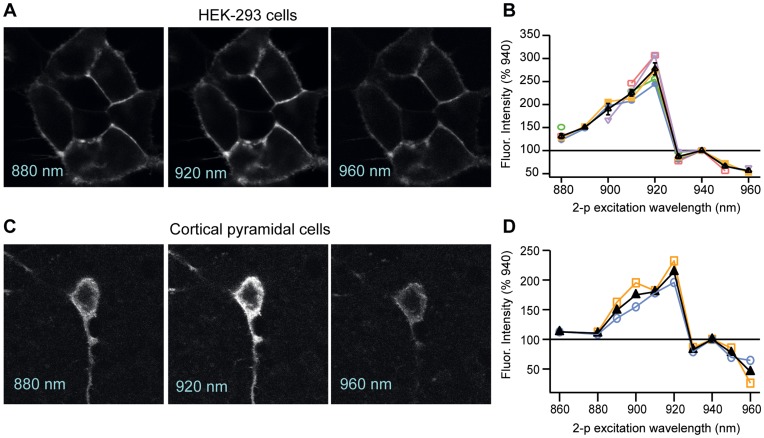
2P excitation spectra for DiO in DPA. A. 2P images of HEK-293 cells labeled extracellularly with DiO at excitation wavelengths of 880, 920 and 960 nm. **B.** Plot of DiO fluorescence intensity across 2P excitation wavelengths in HEK cells (n = 5), normalized to fluorescence obtained at 940 nm. Colored lines and points represent the plots of the individual cells; the black line is the average across cells. **C.** 2P images of a L5 cortical pyramidal cell patch-labeled with DiO at excitation wavelengths of 880, 920 and 960 nm. **D.** Plot of DiO fluorescence intensity across 2P excitation wavelengths in two L5 pyramidal cells, normalized to fluorescence obtained at 940 nm. Laser power, measured at the back of the objective, was held constant across all wavelengths.

Electrophysiological and optical signals were aligned on the peak of the electrically recorded AP prior to averaging. Peak amplitudes and the full widths at half maximum value (FWHM) were calculated from the averaged traces. For experiments without an electrophysiological recording, traces were either aligned on the peak value of fAPs, or in the case of subthreshold signals, on the time of synaptic stimulation. SNR was calculated individually for each cell from the peak of the fluorescence transient elicited by an AP divided by the average standard deviation of baseline fluorescence over a 5-ms period before stimulation.

Mean values are indicated with ± standard error of the mean (SEM). We used JMP (SAS) software for statistical analyses. Linear correlations were examined by performing a linear fit to data and calculating the coefficient of determination (R^2^), F-statistic and a 95% confidence interval. After checking for equality of variance, two-tailed paired or independent-samples t-tests were performed to compare means between two groups. Otherwise, where indicated, one or two-way ANOVAs were performed, followed by Tukey-Kramer pairwise comparisons if the ANOVA yielded a significant result. *’s indicate statistically significant differences (P<0.05).

**Figure 4:**
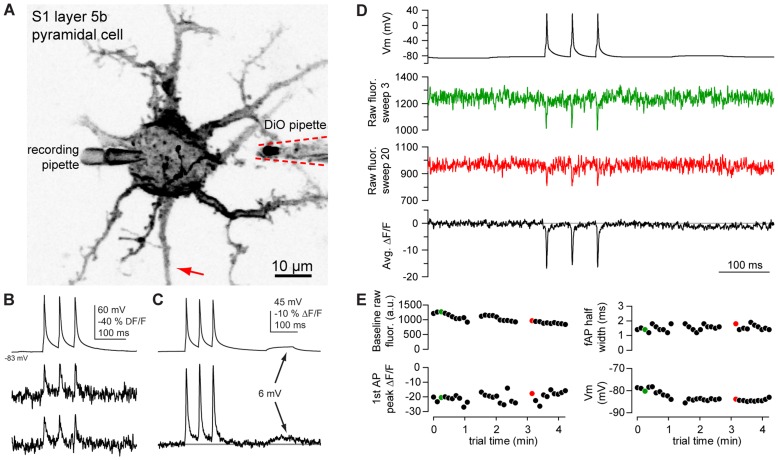
2P excitation of DiO/DPA enables AP detection in a L5 pyramidal cell. A. Negative image of a pyramidal cell membrane labeled with DiO. Dashed lines outline the DiO iontophoretic pipette, withdrawn from the soma after iontophoresis. **B.** Individual fAPs detected using DiO/DPA in the axon initial segment (arrow in A). The top trace is a somatic whole-cell recording of membrane voltage (Vm), and the middle and bottom traces are successive single trials. Fluorescence traces were not inverted. **C**. Average of 50 trials showing fAPs and a DiO/DPA optical response to a 6.8 mV depolarization. Data was corrected for photobleaching (<2% decrease ΔF/F over length of trace). **D.** Vm (top), single trial optical recordings from near the beginning (green) and end (red) of 30 trials, and an average of all 30 trials (bottom trace) from another pyramidal cell. Images were made on soma membrane. No photobleaching correction was necessary in this cell. **E.** Summary of baseline fluorescence, half width and peak ΔF/F of 1st fAP in the train, and baseline Vm, captured in 3 sets of 10 trails. Colored data points correspond to values measured from individual sweeps in D. Note that values were calculated from unfiltered data.

## Results

### Whole-cell DiO Loading Enables Reliable Optical Detection of Single APs and Bursts

Prior work using the 2cVoS combination of DiO and DPA demonstrated that robust voltage-dependent optical signals could be obtained in Purkinje cells of acute brain slices following labeling in a cell-attached configuration and repatching with a second, dye-free pipette [14]. To further simplify patch pipette-based loading procedures, we tested whether cells could be effectively labeled in whole-cell mode. Rather than using dimethylformamide as a solvent for DiO, we used DMSO and diluted this stock into the internal solutions to yield 0.05–0.2% final DMSO concentration. This approach allowed cells to be easily patched and rapidly labeled (<15 minutes) in whole cell configuration (Figure 1A). Such recording conditions often resulted in more extensive and brighter staining of the cell body, axon, and proximal dendrites and these recordings were stable for tens of minutes (Figure 1B-1E). By parking the laser-illuminated excitation spot at various locations within labeled neurons we were able to observe large (>15%) and rapid optical changes, corresponding to quenching of DiO fluorescence (transients are inverted to match the electrical convention for depolarization), in response to single APs and complex spike-like bursts [14]. In Purkinje cell somata, AP-induced DiO/DPA fluorescence transients markedly decremented with distance into the dendrites as expected based on the lack of voltage-gated sodium conductance in Purkinje cell dendrites (Figure 1D). These data demonstrate a more straightforward whole-cell approach which labels proximal membrane compartments quickly and effectively. In many circumstances such an approach will yield excellent results; however, the more laborious two-pipette labeling method will typically result in a higher ratio of plasma membrane to internal staining. As presented below, this ratio is predictive of signal size in most large neurons.

**Figure 5:**
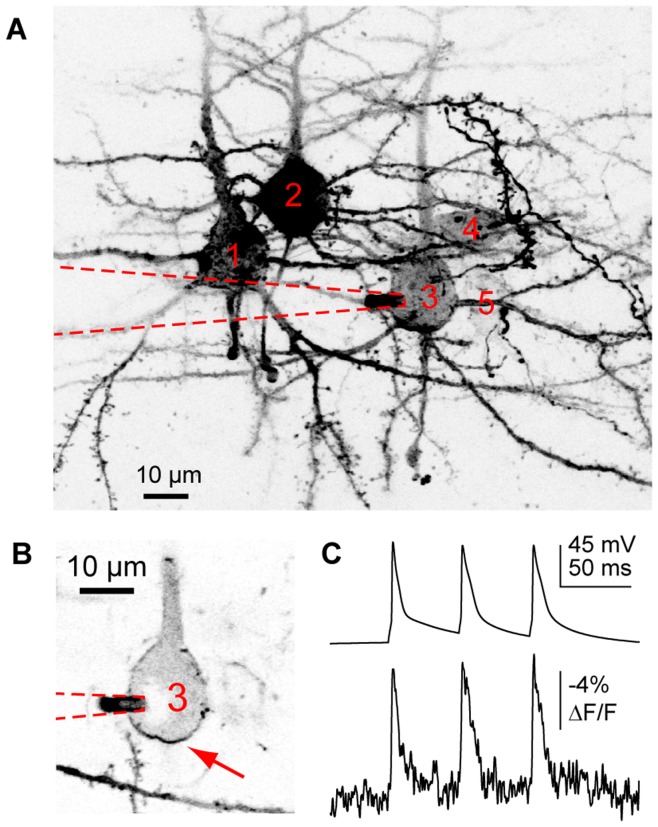
Iontophorektic loading of DiO/DPA in multiple L5 pyramidal cells. A . Compressed Z-stack of negative images of 4 pyramidal cells (indicated as cells 1,2,3, and 5) and 1 interneuron (cell 4) labeled with DiO via iontophoresis. A whole-cell recording was subsequently made from pyramidal cell 3. The red dashed line outlines the whole-cell pipette. **B**. Single optical section detailing the optical recording site (arrow) and whole-cell pipette location (red dotted line) on pyramidal cell 3. The cell was patched 40 minutes after loading. **C.** Average of 40 trials showing the electrical (upper trace) and optical (lower trace) voltage measurements from the cell in B.

### Membrane-specificity of DiO Labeling Correlates with Signal Size

Specificity of membrane labeling is a significant factor in VSD performance [14], [19]. To understand the relationship of this critical factor to signal size we set out to quantify the relationship between membrane labeling and fAP amplitude in Purkinje and hippocampal CA1 pyramidal cells. Cells were loaded at room-temperature (RT, 25–26°C) in whole-cell configuration as described above and the patch pipette was removed after attaining the whole-cell configuration. Confocal images of DiO fluorescence were then obtained before recording of fAPs, which were elicited by current injection through a second patch pipette.

**Figure 6:**
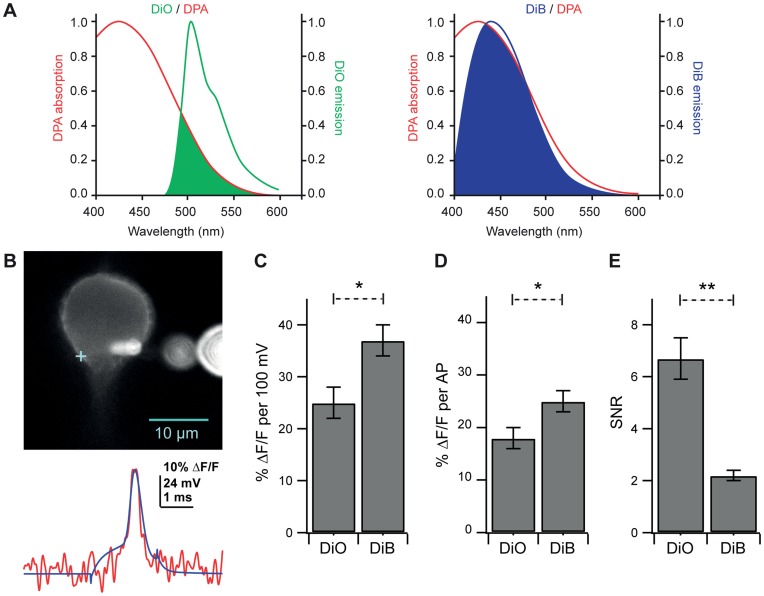
A blue-shifted fluorescence donor increases fAP amplitude but exhibits a decreased SNR. A. Single-photon fluorescence emission spectra of DiO (left, green line) and DiB (right, blue line) overlaid with the excitation spectrum of DPA. Overlap between the emission spectra of the fluorescence donors and the FRET acceptor (DPA) is depicted by the shaded areas of each plot. Note the greater overlap between the emission spectrum of DiB and the excitation spectrum of DPA. **B.** Upper: Confocal image without pinhole of a Purkinje cell patch-labeled with DiB and illuminated with 375 nm laser light. The cross indicates optical recording location. Extracellular fluorescent orbs are out-of-focus dye crystals in the patch pipette. Lower: Optical trace (red) of averaged (12 traces) voltage measurements overlaid with average of simultaneously recorded APs (blue trace) evoked by brief current pulses. **C.** Comparison of % ΔF/F per AP in cells loaded with DiO (18±2% ΔF/F, 488 nm illumination) and cells loaded with DiB (25±2% ΔF/F, 375–405 nm illumination). **D.** Comparison of % ΔF/F per 100 mV in cells loaded with DiO (25±3% ΔF/F) and cells loaded with DiB (37±3% ΔF/F). **E.** Comparison of SNR between cells loaded with DiO (6.7±0.8) and cells loaded with DiB (2.2±0.2). Bars in **C-E** represent the mean ± SEM.

To obtain an estimate of membrane-specific DiO labeling we measured fluorescence in individual neurons across a 1-µm-wide line profile bisecting the cell body (Figure 2A-2B). From the line profile we calculated the following three parameters: (1) “edge” fluorescence; (2) “internal” fluorescence and (3) “relative edge fluorescence”. We defined “edge fluorescence” as the average of the two peaks of maximal fluorescence observed near the plasma membrane on either side of the cell. We defined “internal fluorescence” as the average of 2-µm segments along the line profile, inside the cell and away from the membrane-associated fluorescence peaks. In both Purkinje and hippocampal CA1 pyramidal cells, which often have large visible nuclei that exclude the dye, care was taken to measure internal fluorescence in a region of the line profile excluding the nucleus (shaded region, Figure 2A and 2B). Finally, "relative edge fluorescence” was calculated as the ratio of edge to internal fluorescence in each individual cell.

**Figure 7:**
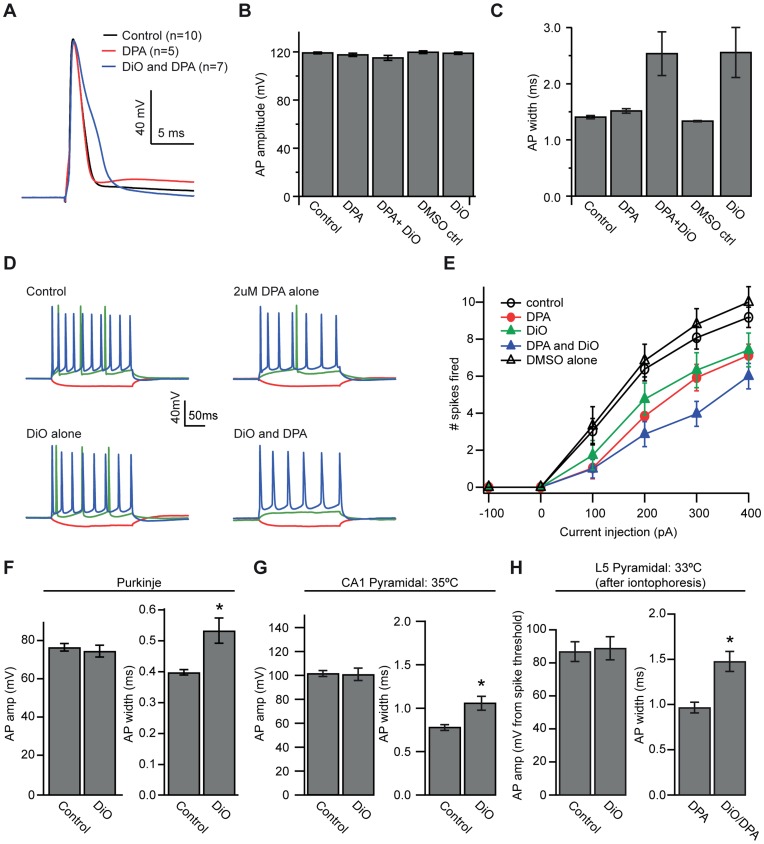
DiO labeling of neuronal membrane alters AP properties. A. Sample AP waveforms from three different hippocampal pyramidal cells recorded in control solutions (black), in 2µM DPA (red) and in DPA and DiO together (blue). **B.** AP amplitude from hippocampal pyramidal cells recorded in control solutions, DPA alone (2 µM), DPA+DiO (2 µM, 1.5 µM, respectively), vehicle control (0.2% DMSO in internal solution), and DiO alone (3 µM). **C.** AP width for the five experimental groups showing a 77% increase in AP width due to DiO. **D.** Sample of individual cells’ responses to pulses of -100 (red), +100 (green) and +400 (blue) pA current injection under control conditions, DPA alone, DiO alone and DPA+DiO together. **E**. Plot displaying number of spikes fired for all 5 groups in response to 200-ms pulses of current injection ranging from -100 to +400pA. **F.** Comparison of AP amplitude (left) and AP width (right) with and without dye in cerebellar Purkinje cells shows a smaller (34%) broadening of the AP in the presence of DiO. **G.** Comparison of AP amplitude (left) and AP width (right) with and without DiO in CA1 pyramidal cells recorded at 35°C shows a 35% increase in width and no difference in amplitude due to DiO. **H.** Comparison of AP amplitude (left) and AP width (right) in cortical pyramidal cells before and after iontophoresis reveal a 53% increase in AP width after dye loading. Bars represent mean ± SEM.

Scatter plots with fAP amplitude versus relative edge fluorescence were then constructed for populations of individual cells. Linear regression analyses revealed a significant correlation between relative edge fluorescence and fAP amplitude in Purkinje (R^2^ = 0.916, F = 75.95, p<0.0001, Figure 2C) and pyramidal cells (R^2^ = 0.632, F = 8.576, p = 0.033, Fig. 2D). These data demonstrate that in large cells the variability in apparent dye labeling of plasma membrane correlates with the fAP size, supporting the conclusion that labeling effectiveness influences the relative change in fluorescence generated by 2cVoS.

**Figure 8:**
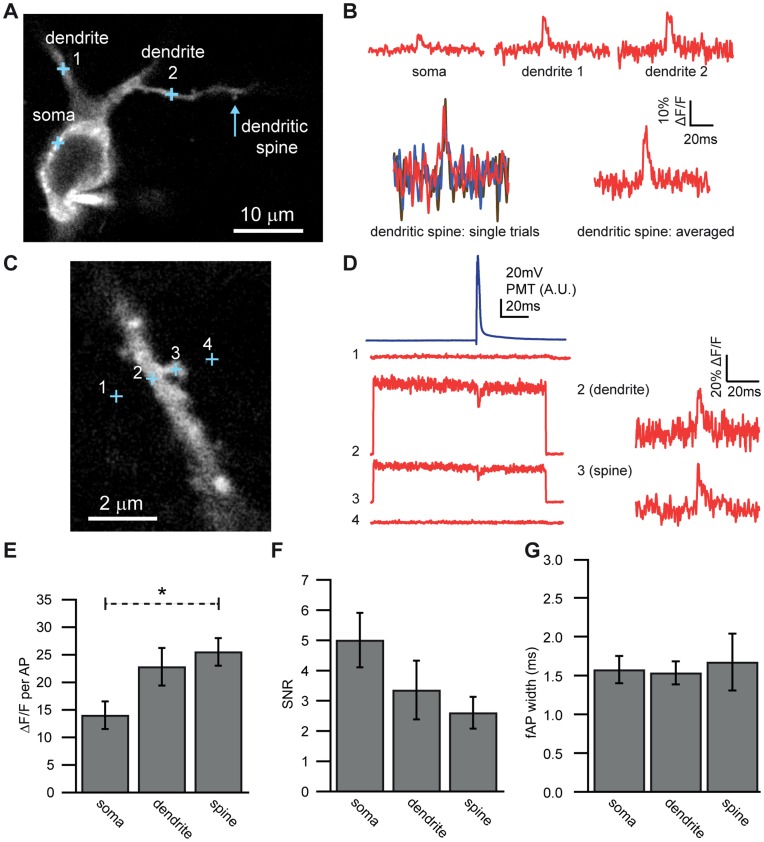
Single-trial optical detection of APs from CA1 pyramidal cell dendritic spines. A. Confocal image of CA1 pyramidal cell and detection spot locations where optical measurements were made (crosses). **B.** Averaged optical traces (in red) recorded from soma (10 traces), primary dendrite (13 traces), secondary dendrite (11 traces) and dendritic spine (11 traces). Blue lines represent electrically recorded APs. In the bottom left is an overlay of three single-trial optical traces recorded from the dendritic spine with a clearly identifiable fAP. **C.** Confocal image of a different CA1 pyramidal cell basal dendrite and recording spot locations. These experiments were conducted using the confocal pinhole. **D. Upper left, blue:** Electrical recording of a somatic AP. **Below left, red:** Averaged, unprocessed fluorescence traces obtained from two points off of the dendrite (point one: 12 traces, and point four: 12 traces), one point on the dendrite (point 2∶17 traces) and one point on the spine (point 3∶24 traces). Note that no significant fluorescence or signal is obtained at points adjacent to the dendritic spine. **Right:** Optically recorded fAPs normalized to baseline fluorescence recorded in the dendrite (32% ΔF/F max, average of 17 traces) and spine (35% ΔF/F max, average of 24 traces) from the cell pictured in 9C. **E.** fAP amplitude compared in soma (n = 10) dendrites (n = 8) and spines (n = 5). **F** SNR compared in soma dendrites and spines. **G**. fAP width compared in soma dendrites and spines. Bars in E-F represent mean ± SEM.

Strong membrane labeling in cerebellar granule cells, a smaller cell type, was consistent with these conclusions. Due to the small size of granule cells, we were not able to discern non-nuclear cytoplasm, precluding reliable estimates of membrane-specific labeling. This is likely due to limitations in the optical resolution and the high degree of membrane curvature. Nevertheless, the plasma membrane labeling in cerebellar granule cells appeared bright and with high contrast, yielding larger fAPs (40±5%, n = 10) than either Purkinje cells (15±3%, n = 9) or hippocampal CA1 pyramidal cells (15±2%, n = 7). Much like for axons, we reason that the strong fluorescence labeling of cerebellar granule cells is specific to the plasma membrane and thereby accounts for the larger ΔF/F. Taken together these data also suggest that relative edge fluorescence greater than 2.5 is a good criterion for optimizing DiO/DPA sensitivity. Consistent with this, when cells that do not exceed this threshold are excluded, we calculate a ΔF/F of 19±1% for fAPs in that subset of Purkinje and pyramidal cells (n = 11).

**Figure 9:**
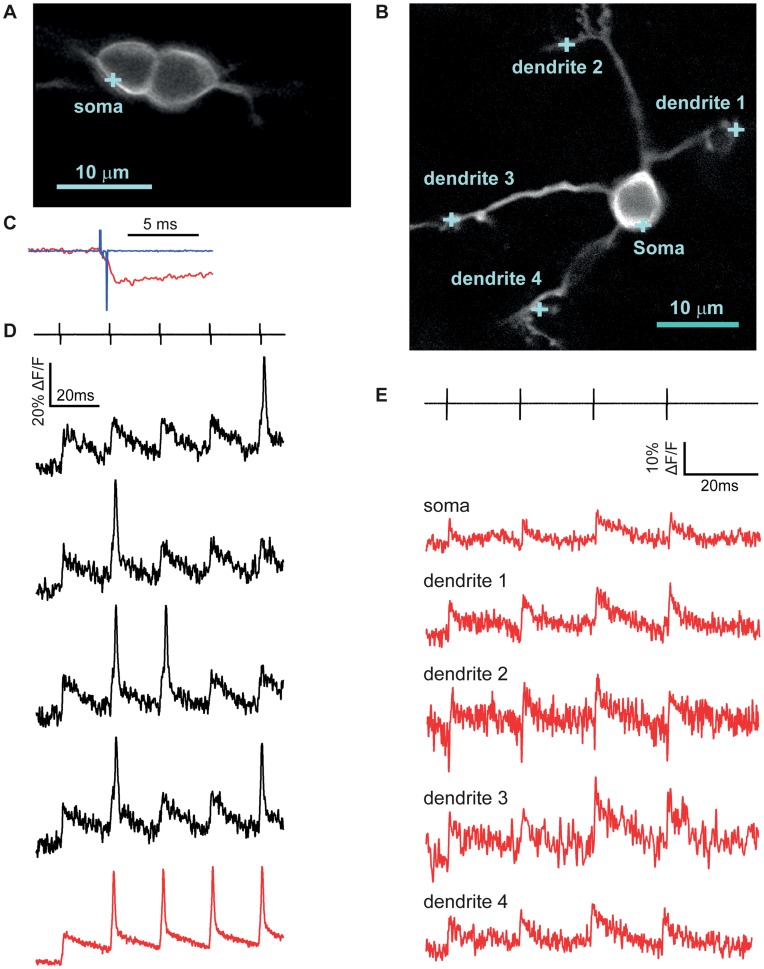
Single-trial detection of EPSPs in cerebellar granule cells. A. Confocal image of two GCs patch-labeled with DiO. The blue cross represents the illumination spot on the somatic membrane where the optical measurement was taken. **B.** Confocal image of a different granule cell patch-labeled with DiO. Blue crosses mark the points where optical measurements of voltage were obtained. **C.** Expanded view of fluorescence quenching upon depolarization (f-EPSP) by extracellular stimulation of putative single mossy fiber (f-EPSP), overlaid with the stimulus artifact. Note the decreasing slope of the fluorescence change after the end of the stimulus. **D.** Four single-trial optical recordings (black) of mixed f-EPSPs and fAPs measured from the cell depicted in panel A. The black lines above represent the electrical stimulation (5 pulses at 50 Hz) used to activate single mossy fiber inputs. The red trace below is the average of 5 individual traces where a subthreshold response was followed by 4 fAPs. **E.** Averaged optical responses (red traces) to a 50 Hz 4-pulse train of stimulation (top, black lines) obtained from the soma (5 traces), dendrite #1 (5 traces), dendrite #2 (4 traces), dendrite #3 (3 traces) and dendrite #4 (7 traces) of the cell displayed in panel B.

### 2P Excitation of DiO/DPA

Recognizing the importance of imaging cells deeper in tissue, we next tested whether DiO (in the presence of 2 µM DPA) is effectively excited by 2P excitation. We found that in HEK cells, 2P excitation of DiO yielded robust membrane fluorescence (Figure 3A) that varied as a function of excitation wavelength (Figure 3B). The brightest fluorescence signals occurred over a narrow part of the 2P excitation spectrum, with a peak fluorescence emission in response to excitation at 920 nm. The fluorescence intensity decreased by 2-fold for wavelengths less than 900 nm and greater than 930 nm (n = 5, fluorescence normalized to 940 nm). Cortical pyramidal cells exhibited a similar peak at 920 nm and similar fall-off around this peak (Figure 3C-3D, n = 2), indicating that the 2P excitation properties of DiO are similar across cell types.

**Figure 10:**
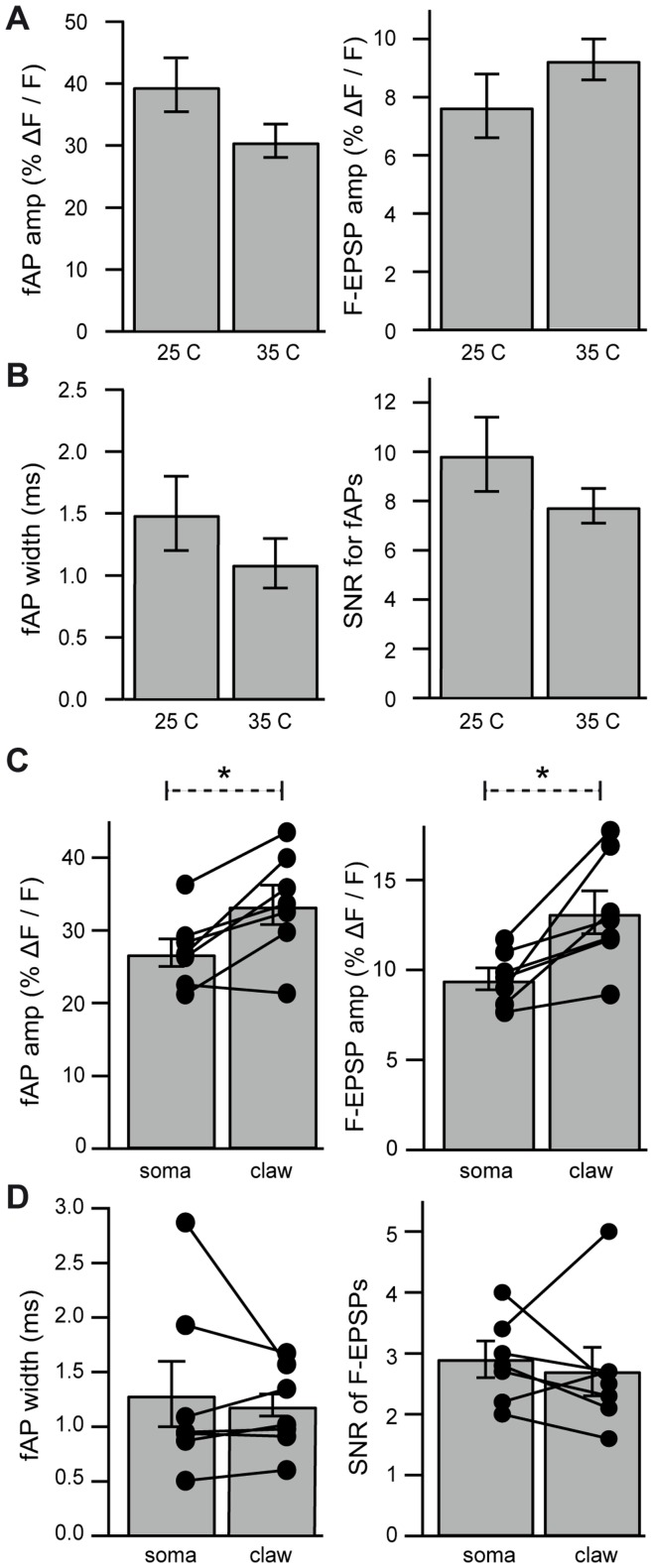
Properties of fAP and f-EPSP recorded from subcompartments of granule cells. A. Left: Comparison of fAP amplitudes recorded from granule cell somata at 25°C (n = 12) and 35°C (n = 14). **Right:** Comparison of f-EPSP amplitudes recorded from somata of granule cells at 25°C (n = 8) and 35°C (n = 10). **B. Left:** Comparison of fAP width at 25°C and 35°C. **Right:** Comparison of SNR at 25°C and 35°C. **C. Left:** Pair-matched comparisons of optically recorded fAP amplitude between the soma and dendritic claws of granule cells (n = 7) recorded at 35°C. **Right:** Pair-matched comparisons of optically recorded f-EPSP amplitude between the soma and dendritic claws of granule cells (n = 7). **D. Left:** Pair-matched comparisons of fAP widths between the soma and dendritic claws. **Right:** Pair-matched comparisons of SNR of f-EPSPs between the soma and dendritic claws.

Guided by these data, we then measured AP-evoked fluorescence transients from DiO labeled cortical pyramidal neurons in the presence of 2 µM DPA. Using whole-cell current-clamp methods, we evoked APs via somatic current injection and performed 2P excitation using parked beam (2P spot detection) recordings of DiO/DPA fAPs in the somata and proximal regions of the axon (Figure 4A). At near physiological temperatures (33°C) somatic fAPs (Figure 4B) were easily detectable, yielding somatic fluorescence transients of –18.0±8.2% ΔF/F (n = 3) and axonal transients of -23.8±3.2% (n = 4), values comparable to single-photon recordings. In some cases the SNR was sufficient to detect AP evoked responses in single trials (Figure 4B) or even subthreshold depolarizations of less than 10 mV upon signal averaging (Figure 4C).

To determine whether the SNR achieved with 2P excitation of DiO/DPA produced significant toxicity, we examined the raw fluorescence traces early and late in a series of stimulation trials (30 stimuli delivered in 250s). We observed very little dye photobleaching during the illumination early and late in the experiment (Figure 4D, green and red trace, respectively), and in the average trace (black). We found the resting membrane potential of the electrical recording to be stable over those 30 traces, with a small decline in the baseline fluorescence, and no change in the peak ΔF/F or width of the fAP (Figure 4E). We attribute the decrease in baseline fluorescence over time to the diffusion of dye throughout the entire neuron (see also [14]). In some cells this protocol could be performed at multiple locations within a ∼10 µm axonal segment allowing up to 150 trials without detectable photodamage. Notably, under these conditions we had sufficient SNR to detect a small voltage deflection of ∼5 mV in the averaged trace (Fig. 4C). These findings demonstrate that optical voltage detection with DiO/DPA 2cVOS is possible using 2P excitation, allowing the robust and stable recording of membrane potential from neuronal processes deep (>100 µm) in scattering tissue within single trials.

### Iontophoretic DiO Labeling of Cortical Pyramidal Cells and fAPs Recorded Using 2P Excitation

In our experience the most difficult step in implementing this technique is effective labeling of neurons in a directed manner. While cell-attached and whole-cell labeling can be reliable, they are laborious methods that require separate pipettes for each labeled cell. In order to improve the ease and efficiency of DiO labeling, we examined a number of alternative loading procedures and found that iontophoresis proved effective at targeted extrusion of the positively charged DiO molecules. To label single neurons we filled patch pipettes with DiO dissolved in 100% ethanol, and positioned the pipette at the surface of the neuronal cell body of interest, visualized simultaneously using laser scanning Dodt contrast and 2P fluorescence imaging. Brief positive current pulses (0.5 to 10 nA) labeled neurons robustly and extensively throughout their dendritic and axonal processes (Figure 4A and 5D). The main challenge in labeling with this method was clogging of the pipette tip with dye particles. Often a larger current could dislodge the particles, but we found that if we were scrupulous about maintaining dye solubility we could minimize clogging (see Methods section). This was accomplished by keeping the DiO/DMSO stock solution heated to 50°C, and regularly vortexing and sonicating the DiO-containing pipette solution. Rapid, successive labeling of multiple cells from the same slice was straightforward with this approach (Figure 5A). After labeling up to 5 cells, optical detection of somatic APs was still possible (Figure 5B,C). Such use of iontophoresis greatly simplifies neuronal labeling and coupled with the excellent 2P performance suggests that 2cVoS will be useful for monitoring membrane voltage from multiple neurons within a network.

### A Blue-shifted Fluorescence Donor (DiB) Increases 2cVoS Sensitivity but Reduces SNR

FRET theory dictates that the efficiency of energy transfer in FRET depends on the spectral overlap between donor and acceptor [20], [21]. Thus, if the 2cVoS mechanism relies on FRET, blue-shifted donors should increase the ΔF/F because they will overlap more effectively with the blue absorbance spectrum of DPA [22], [23], [24], [25]. With this in mind we examined whether a blue-shifted carbocyanine dye, DiB (Figure 6A), would exhibit a greater sensitivity than DiO/DPA. To compare the two fluorescence donors DiO and DiB, we performed current clamp recordings of APs with DiO or DiB in the patch pipette solution (Figure 6B). We found that DiB/DPA produced a ΔF/F per AP (25%; n = 9; p = 0.040) and ΔF/F per 100 mV (37%; p = 0.013) larger than those produced by DiO/DPA (Figure 6C,D; n = 10). However, the increase in sensitivity was accompanied by a three-fold reduction in SNR (Fig. 5E, p = 0.0003). These data confirm that a FRET-based mechanism is at least in part responsible for the voltage-sensitive optical signals observed using the dye-DPA 2cVoS combination. Despite this enhancement, the lower SNR produced by DiB/DPA renders DiO/DPA the more preferable 2cVOS.

### Modest Effects of DiO but not DPA on AP Width in Hippocampal and Cortical Pyramidal Cells

Previous work has demonstrated that 2 µM DPA in external solution does not change the characteristics of single APs in Purkinje cells but DiO effects were not measured [14]. To examine in more detail whether DiO and DPA exert effects on neuronal physiology, we recorded single APs and bursts of APs from hippocampal CA1 pyramidal cells at 25°C in the following conditions: (1) in control solutions containing no DPA, DiO or DMSO (n = 10), (2) in 2 µM DPA alone (n = 5), (3) labeled with 0.2% (3 µM final) DiO- DMSO (n = 5), (4) labeled with DiO in DPA (n = 7), and (5) internally loaded with the dye vehicle (0.2% DMSO) alone (n = 5). Dye and vehicle loading (conditions 3–5) were performed by brief on-cell patch-loading, and cells were then re-patched; cells in control solutions or DPA alone (conditions 1 and 2) were only patched once. We measured the amplitude and width of single APs evoked by 0.5 ms current injections, as well as the number of spikes fired in response to 200 ms current injections. In neurons loaded with DiO and DPA, APs and bursts were measured before obtaining spot-measurements to ensure that effects were not related to repeated imaging.

AP amplitude was not affected by either DiO or DPA (Figure 7B; 2 way ANOVA: DiO, F = 1.06, p = 0.315; DPA, F = 4.04, p = 0.056; DiO/DPA interaction, F = 0.68, p = 0.417). However, we were surprised to find that AP width was significantly prolonged by DiO but not DPA (Figure 7C; DiO, F = 16.29, p = 0.0005; DPA, F = 0.02, p = 0.895; DiO/DPA interaction; F = 0.06, p = 0.8083). The broadening by DiO slowed the falling phase of the AP without a significant effect on the peak, leading to an increase in half width from 1.45±0.03 ms in control cells to 2.60±0.45 ms in the presence of DiO alone (Figure 7A-C). We then tested whether this AP broadening could affect burst firing induced by 200-ms current injections. As shown in Figure 7D and E, we did find that either DiO alone (F = 5.22, p = 0.032) or DPA alone (F = 7.33, p = 0.013) reduced the number of spikes fired in response to a 400 pA current injection, without any significant interaction between the two (F = 0.07, p = 0.798). We observed no significant difference in either AP amplitude (p = 0.67) or AP width (p = 0.17) between cells recorded in control solutions and cells that had been patch-loaded with DMSO alone. We also observed no effect of patch-loading with DMSO alone on the number of spikes fired in response to a 200-ms 400-pA pulse of current (p = 0.42), indicating that patch-loading and subsequent re-patching are not responsible for DiO effects on AP width. Similar results were obtained in recordings from Purkinje cells recorded at 25°C (Figure 7F), although the degree of AP broadening in the presence of DiO was smaller than for CA1 pyramidal neurons (DiO half width, 0.53 ms, n = 9; control half width 0.40 ms, n = 9, p = 0.006).

We found that this effect of DiO was not only cell-type specific but also temperature dependent. As compared to 25°C, broadening of CA1 pyramidal cell APs was much less at the near physiological temperature of 35°C, (0.79 ms in controls, n = 7; 1.07 ms in DiO, n = 6, p = 0.013; Figure 7G). Similarly, in cortical pyramidal cells at 33°C, DiO iontophoresis led to a 50% increase in AP width (Figure 6H) to 1.48 ms compared to 0.97 ms before loading (n = 7, p = 0.003). AP width was not significantly altered by sham (100% ethanol) iontophoretic loading (0.91 ms before, 0.93 ms after, n = 3), nor were other AP properties affected, indicating that the iontophoresis itself does not affect AP properties. The results presented here suggest that the effects of DiO on AP width are cell-type specific and are reduced at physiological temperatures. Nevertheless, they identify subtle pharmacological effects of DiO on neuronal function which must be considered when performing experiments with DiO/DPA.

### Single-trial bAP Recordings in Dendritic Spines at Physiological Temperatures

To determine whether DiO/DPA is capable of reporting membrane potential signals under physiological conditions from small subcompartments of dendrites we examined dendritic spines in CA1 pyramidal cells at 35°C. Current injection through the somatic whole-cell pipette trigged APs which backpropagated into the dendrites and spines. Placement of one-photon (1P) laser spots on different subcompartments allowed us to observe robust fAPs not only at the soma, but also in nearby dendrites and dendritic spines, even in single traces (Figure 8A-8D). The fAP amplitude was significantly different across different neuronal compartments (one-way ANOVA, F = 4.28, p = 0.028), with fAPs in spines being significantly larger than fAPs at the soma (Figure 8B & E; spine: 25.6±2.5% ΔF/F, n = 5; soma: 14.1±2.5% ΔF/F, n = 10; p = 0.046). This difference may be in part due to differences in plasma membrane versus intracellular labeling and/or higher surface to volume ratios as suggested by the data in Figures 1 and 2. The SNR, however, was lower in the spine, likely due to the low fluorescence intensity resulting from the small membrane area within the detection volume (Figure 8F). The fAP width was not different between compartments (Figure 8G). These results demonstrate that DiO/DPA can readily exhibit optical responses to electrical activity in dendrites and spines in single trials at near physiological temperatures.

### The DiO/DPA 2cVoS Reports Subthreshold Synaptic Depolarizations in Single Trials

By exploiting the high sensitivity of DiO/DPA, especially under strong membrane labeling conditions, we explored the feasibility of detecting synaptically-elicited, subthreshold depolarizations. Granule cells were briefly patch-loaded with DiO (Figure 9A,B) in the presence of 2 µM DPA in the bathing solution, and excitatory afferent synaptic inputs were stimulated with an electrode placed near labeled dendritic claws. Stimulus intensity was adjusted to just above threshold for detecting an optical synaptic response (f-EPSP) in the soma (see Methods). Using this approach, subthreshold synaptic inputs were discernible in single trials and were clearly distinguishable from suprathreshold synaptic inputs that occurred stochastically in response to the stimuli (Figure 9D). Direct depolarization of the cells could be ruled out by comparison of the time courses of the responses and of the electrical artifacts measured by the dye loading pipette left nearby in the bath (Figure 9C). Many cells fired occasional APs in response to trains of synaptic stimulation, regardless of stimulation strength (15 out of 18 cells total showing synaptic responses also fired fAPs). In other cells, subthreshold depolarizations were detected, but fAPs were not apparent.

The large SNR allowed us to test how a synaptic input on one dendritic claw influences membrane potential at the soma and in the other dendritic claws. The granule cell shown in Figure 8B possessed four dendritic claws in a single plane of confocal imaging. Stimulation of dendrite 2 (see negative shock artifact) gave rise to clearly detectable fluorescence signals in the soma and each of the dendritic claws, shown as signal averages of between 3 to 7 traces, obtained at each location indicated in Figure 9B. These f-EPSPs were all greater than 10% ΔF/F and similar among dendrites (12.3%, 11.5%, 15.9% and 11.0% for dendrites 1,2,3 and 4, respectively) consistent with little voltage decrement from the site of synaptic stimulation and confirming simulations and somatic electrode recordings showing that mature granule cell somata and dendrites comprise a single electrotonic compartment [26], [27].

Figure 10 summarizes sub- and suprathreshold signals from granule cell somata and dendrites elicited by synaptic stimulation. Somatic subthreshold signals (f-EPSPs) were similar at room and physiological temperatures (25°C: 8±1% ΔF/F; 35°C: 9±1% ΔF/F, p = 0.231, Figure 10A). Suprathreshold signals were also similar at the two temperatures (25°C: 40±4% ΔF/F, n = 12; 35°C: 31±3% ΔF/F, n = 14, p = 0.093). As shown in Figure 10B, no overall difference was observed between the width of fAPs or SNR at 25°C (9.9±1.5) or 35°C (7.8±0.7). We also compared the DiO/DPA signal size observed in pair-matched recordings of fAPs and f-EPSPs from the soma and dendritic claws of individual granule cells at 35°C. We found that fAPs were larger when measured in the claws (34±3% ΔF/F) than when measured at the soma in the same cells (27±2% ΔF/F, p = 0.009; Figure 10C). We considered the possibility that AP invasion of the dendrite might increase in size as it approaches a sealed-end cable, as modeled previously [28], but we did not observe a change in fAP width measured in dendrites (1.2±0.1 ms) relative to those in the soma (1.3±0.3 ms, p = 0.485; Figure 10D). We therefore reasoned that even for granule cells, spatially inhomogeneous levels of membrane labeling may account for the larger AP in dendritic hands versus somata. Indeed, the f-EPSP amplitudes from pair-matched cells were also larger in dendritic claws (13±1% ΔF/F) than at the soma (10±1% ΔF/F, p = 0.012; Figure 10C). The SNR of f-EPSPs was similar for both the soma (2.9±0.3) and in the dendrites (2.7±0.4, p = 0.660). Taken together, these findings show that when membrane labeling is favored, as in granule cells, DiO/DPA is capable of detecting subthreshold synaptic responses in thin dendritic processes.

## Discussion

Optical voltage sensors are essential tools for answering otherwise unapproachable questions about information processing in neuronal networks. We have systematically characterized the behavior of the DiO-DPA 2cVoS [14] in several different types of neurons in acute slices of mouse brain. This manuscript describes technical strategies for optimizing its sensitivity and for facilitating directed labeling of individual neurons; we also report shortcomings of the method and suggest ways to minimize their impact. Finally we demonstrate that the method works in a wide variety of neuronal types and that it can be implemented with 2P illumination. To highlight the performance of the method we show that back propagated-AP signals can be detected in single trials in dendritic spines of CA1 pyramidal neurons and that subthreshold synaptic potentials can be recorded in single trials in cerebellar granule cells. Such benchmarks for kinetic properties, signal size, and SNR qualify this as among the best optical voltage sensor systems at this time.

### Determinants of DiO Labeling and its Influence on DiO/DPA Sensitivity

Our survey of several neuronal types (Purkinje neuron, cerebellar granule cell, CA1 pyramidal cell, cortical pyramidal cell) indicated that the level of plasma membrane relative to internal labeling is a strong determinant of peak ΔF/F per AP. In certain cells (e.g. granule cells) we regularly observed ΔF/F values exceeding 30% (Figures 2, 9, 10). Axons of various cell types also consistently showed large signals as did excitable dendritic compartments of certain cell types such as CA1 pyramidal cells (Figure 8). In both circumstances we hypothesize that the larger ΔF/F values result from greater plasma membrane-specific labeling relative to intracellular fluorescence and/or a higher surface-to-volume ratio. Nevertheless, the SNR is often better at the soma (Figure 8) due to the larger number of photons (N) [29] increasing the SNR by √N [14]]. At physiological temperatures the DiO/DPA 2cVoS is sensitive enough to detect APs in single trials in both somatic compartments and in dendritic spines. Finally, if the experimental goal is to measure APs our findings indicate that focusing on the axon will ensure maximal signal sensitivity.

### Simplified DiO Labeling Using Iontophoresis

The use of iontophoretic loading of DiO is a technical improvement that should simplify the use of the 2cVoS. This loading method seems to produce few deleterious effects on cell health or physiology, while allowing the labeling of multiple cells in quick succession. Iontophoresis has the additional advantage that experimenter-identified cells can be labeled individually. This practicality makes it possible to target cells of a particular type or to screen for multiple cells based on their healthy appearance. These methodological improvements enable voltage recording from multiple neurons within brain tissue, a critical step for neuroscience research if we are to study information flow within neuronal networks.

### 2P Excitation Compatibility

We found that, like certain voltage-sensitive dyes [3], [30], DiO/DPA generates fluorescence signals within acute brain slices that can be readily observed by 2P excitation. However, only a limited number of electrochromic dyes allow 2P excitation [3], [5], [30], [31]. 2P excitation of DiO enables recording from neurons deeper in brain tissue, thereby facilitating recordings *in vivo*. The use of 2P excitation also enables the use of fast acousto-optical device-based random access scanning methods [32], [33], which make possible near-simultaneous detection of DiO/DPA voltage signals at multiple locations, either in different neurons or in several locations within a single neuron.

### Mechanism of DPA Quenching of DiO Fluorescence

In order to validate that DiO/DPA operates via a FRET mechanism, we conducted experiments that substituted a blue-shifted carbocyanine dye (DiB) in place of DiO as a donor. DiB exhibits greater spectral overlap with DPA (Figure 6) which, other parameters being equal, should yield a larger Förster distance and greater FRET efficiency [23], [29], [34]. DiB/DPA yielded ∼50% larger ΔF/F per 100 mV consistent with a FRET-based quenching mechanism for 2cVoS (Figure 6C). This increase is less than the 2-fold increase observed when replacing GFP-based hVoS with a CFP-based hVoS [23], [24]. Potential explanations for this include a reduced extinction coefficient or quantum yield of DiB, internal fluorescence/autofluorescence causing an underestimate of the true increase in ΔF/F per 100 mV, or a FRET-independent mechanism such as collisional quenching between DiO and DPA molecules [6].

Although DiB/DPA exhibited an increased sensitivity, it showed a reduced SNR. This was a result of decreased emitted fluorescence resulting from either: 1) the low intensity UV/near UV light needed to minimize toxicity, and/or 2) the poor photophysical properties of DIB (low extinction coefficient or quantum yield). We also observed higher background fluorescence when exciting with 405 nm laser light as compared to 488-nm illumination, which contributes added fluorescence noise without increased sensitivity. Thus, despite the improved sensitivity, the poor SNR of DiB/DPA fluorescence indicates that DiO is the preferred donor for DPA.

### Effects of DiO on AP Shape

Existing optical methods for monitoring membrane voltage have various drawbacks including direct pharmacological effects on neurotransmitter-gated receptors [35], [36] and prolonged, use-dependent effects on excitability [37], [38]. Although high concentrations of DPA can alter electrical excitability [14], [23], we were surprised that in CA1 and cortical pyramidal neurons DiO had more significant effects on AP shape than 2 µM DPA. The broadening of APs and the reduction in the number of spikes elicited by a long depolarizing current pulse imply that DiO itself can affect ionic conductances that govern AP repolarization. The slower repolarization phase indicates the possibility of an antagonist effect on potassium channels. Indeed, the AP broadening appeared to vary considerably according to cell type and temperature, also suggestive of actions on particular channel types. These pharmacological effects must be taken into account when using DiO/DPA 2cVoS.

The DPA effects on excitability were relatively modest and consistent with those described in the literature [14], [22], [25]. In the present work we provide further evidence (Figures 9 and 10) that synaptic transmission is not impeded by concentrations of 2 µM DPA as has been suggested by studies of *Drosophila* nerve terminals [24].

### Optical Detection of Single EPSPs in Single Trials and APs in Spines

We have taken advantage of the impressive SNR of DiO-DPA to detect single-trial fAPs and f-EPSPs in dendritic spines of CA1 pyramidal cells and dendritic claws of cerebellar granule cells, respectively. High SNR, single-trial, optical signals of back-propagating APs into spines have been recently obtained with electrochromic voltage dyes [3], [4]. Previous work has also demonstrated optical signals from spines upon averaging between 40–160 trials [2], [39]. While measurements of EPSPs have been obtained from larger areas with multi-trial averaging [2], [40], [41], [42], we demonstrate for the first time optical recordings of fast EPSPs in single trials using single voxel recordings from subcellular compartments.

These synaptic responses recorded by DiO/DPA show synaptic integration in granule cells without the disturbances that arise from whole-cell patch electrodes. These data confirm that single mossy fiber inputs can depolarize the granule cell to AP threshold and that synaptic summation from multiple inputs is thus not required [43]. Such minimally invasive single-neuron approaches open the door to studies of synaptic integration in granule cells and in other neurons.

### Conclusions

Together, our findings demonstrate the feasibility of using DiO-DPA for suprathreshold and subthreshold signal detection in small cells and their fine processes in the absence of a recording pipette. The high sensitivity of this method allows clear detection of depolarization in single trials, and the sensitivity turns out to be particularly high in small subcellular compartments. The voltage sensitivity is sufficient to resolve large (20–40 mV) subthreshold depolarizations in single trials, and depolarizations as small as 5 mV with signal averaging. With this level of sensitivity FRET-based voltage sensors are likely to become a crucial component in the neurophysiologist’s toolbox.
